# Is myelo-ablative pretransplant conditioning really myelo-ablative: Implications for radiation and nuclear accidents?

**DOI:** 10.1038/s41409-023-02130-0

**Published:** 2023-11-22

**Authors:** Robert Peter Gale, Hillard M. Lazarus

**Affiliations:** 1 Centre for Haematology, Department of Immunology and Inflammation, Imperial College of Science, Technology and Medicine, London, UK; 2 Department of Medicine, Case Western Reserve University, Cleveland, OH USA

**Keywords:** Cancer, Diseases

Hematopoietic cells are very sensitive to damage from ionizing radiations [1–6]. The estimated 50 percent lethal dose (LD_50_) to humans is 3–4 Gy without supportive care. Doses > 8–10 Gy are thought to cause irreversible bone marrow damage which can only be reversed by a haematopoietic cell transplant.

In the context of transplants high-dose radiation is typically given as a 10–12 Gy single- dose or in a few fractions at dose-rate <10–20 cGy/min. Often radiation is combined with anti-cancer drugs such as cyclophosphamide [7]. These regimens are widely-termed *myelo-ablative*. We briefly discussed the jargon use of *myelo-ablative* previously [8]. In this Commentary we again suggest the term myelo-ablative is wrong and the potential to compromise care of victims of radiation and nuclear accidents. We explain why.

The 1st argument against the term myelo-ablative is the stochastic nature of radiation damage. There cannot be a radiation dose which could kill every hematopoietic stem cell without immediately killing the recipient. The 2^nd^ argument comes from clinical data. In October, 1991 a 34-year-old male operator at an industrial sterilization facility using a ^60^Co γ source in Nesvizh, Belarus was exposed in 1.5 min to an estimated whole-body radiation dose of 11 ± 1.3 Gy (12–15 Gy), with some sites receiving up to 20 Gy [9, 10]. Estimated whole-body dose from computer simulations was 8–16 Gy, from physical dosimetry (electron spin resonance [ESR]), 11–18 Gy ± 20%, and from biological dosimetry, blood cell concentration declines, 9–11 Gy, cytogenetics, 11 ± 1.3 Gy [9, 10], The estimated dose-rate was 5.33–12 Gy/min or 100 times greater than the dose-rate typically used for pretransplant conditioning. He received supportive care, sargramostim and interleukin-3 but no transplant because of anticipated irreversible lung damage. He died 113 days after exposure from pulmonary failure.

**Fig. 1:**
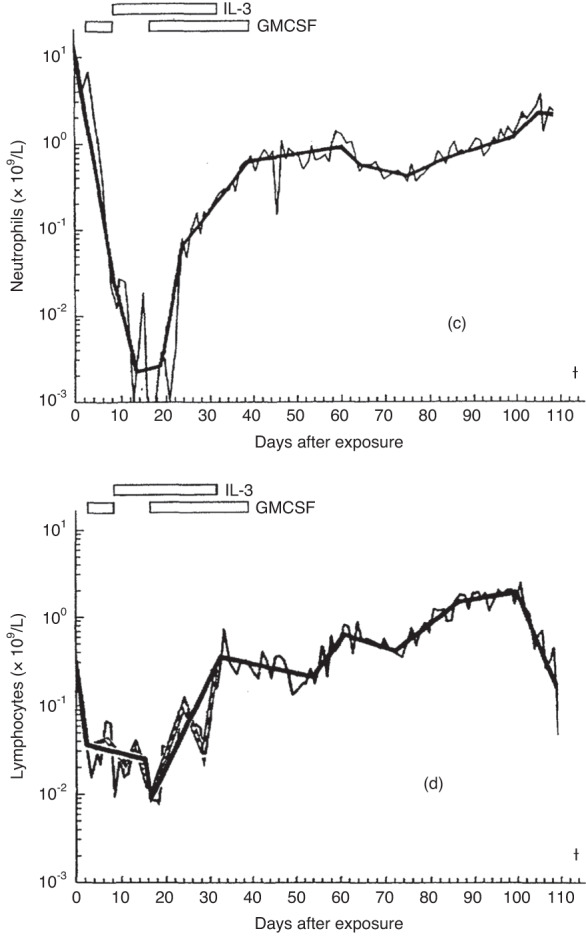
Blood cell concentrations after radiation exposure. Blood neutrophil (**c**) and lymphocyte concentrations (**d**) after exposure.

**Fig. 2:**
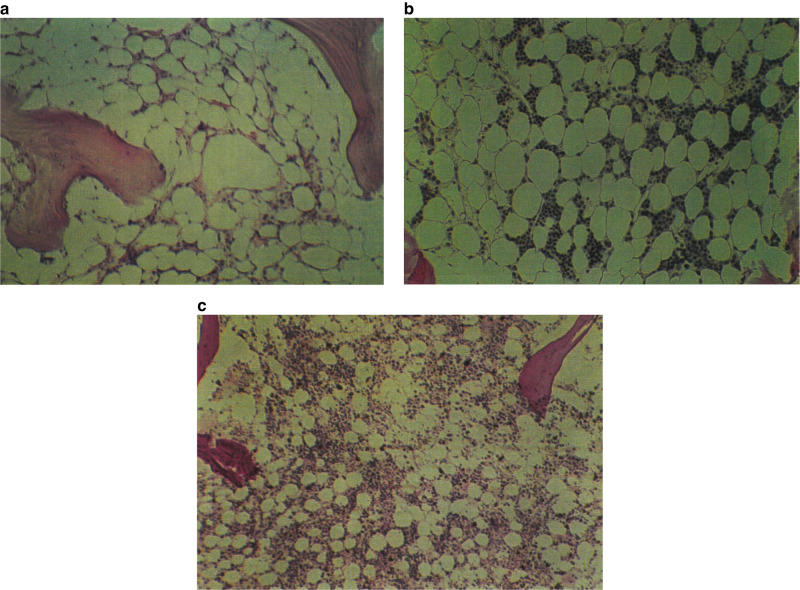
Bone marrow biopsies after radiation exposure. **a** Bone marrow biopsy day 16. Aplasia with only a few macrophages (x100). **b** Bone marrow biopsy day 44. Increase in cellularity (x60). **c** Post-mortem bone marrow day 113. Normal cellularity.

His blood cell concentrations fell immediately after exposure (Fig. 1). At one week a bone marrow biopsy showed severe hypoplasia (Fig. 2a). The blood granulocyte concentrations began to increase on day 23, reaching 0.5 X 10E + 9/L on day 37 and 1.0 X 10E + 9/L on day 60. Bone marrow cellularity began to increase and a bone marrow examination on day 44 after exposure showed improving cellularity (Fig. 2b). A bone marrow examination done on day 113 at autopsy shows near normal cellularity consistent with recovery of blood granulocyte concentrations (Fig. 2c). These data indicate recovery of bone marrow function even after extremely high whole-body dose and dose-rate exposure without a transplant. Clearly this dose and dose-rate, much higher than used for pretransplant conditioning, were not *myelo-ablative*.

Several people exposed to high-dose and dose-rate ionizing radiations after the Chernobyl nuclear power facility accident in 1986 receiving an allogeneic hematopoietic cell transplant recovered autologous bone marrow function after transient engraftment [11]. There are other examples where people with leukemia receiving high-dose radiation (usually 10 Gy in 1 dose or 12 Gy in 6 fractions) followed by an allotransplant are found to have recovery of endogenous hematopoiesis several years later. These data indicate using the term *myelo-ablative* for even very high-dose and dose-rate ionizing radiations is wrong. (We note several of the data we cite are from previously healthy persons receiving no prio or concurrent bone marrow damaging drugs ot a whole-body ionizing radiations).

Humans exposed to acute, high-dose and high-dose-rate whole-body ionizing radiations such as after a radiation or nuclear accident develop hematopoietic acute radiation syndrome (H-ARS) characterized by acute severe myelosuppression with resultant infection, bleeding, and anemia. Exposure to >8 Gy whole-body radiation under these circumstances does not necessarily imply a hematopoietic cells transplant is needed to restore normal bone marrow function [12]. However, such exposures require intensive supportive care. Elsewhere we discuss data in experimental animals including monkeys and data from humans suggesting safety and efficacy of molecularly-cloned hematopoietic growth factors such as sargramostim and filgrastim and possibly eltrombopag [13].

The bottom line is use of the term *myelo-ablative* for radiation-based high-dose pretransplant conditioning regimens is wrong and should be abandoned. A better term would be *intensive* pretransplant conditioning. We accept we are fighting a losing battle but feel obliged to try.
